# Clonal T Cell Proliferation Induced by Acute Anaplasmosis in a Dog

**DOI:** 10.1111/jvim.70233

**Published:** 2025-08-28

**Authors:** Sean R. Teichner, Lisa L. Powell, Allison S. Mazepa

**Affiliations:** ^1^ BluePearl Pet Hospital Golden Valley Minnesota USA

**Keywords:** *Anaplasma phagocytophilum*, flow cytometry, lymphoma, PARR

## Abstract

A 4‐year‐old spayed female Golden Retriever was examined for hindlimb lameness, lethargy, poor appetite, and pyrexia 3 weeks after the removal of numerous ticks. Complete blood count revealed moderate thrombocytopenia and mild lymphopenia. A SNAP 4Dx test was negative *for Borrelia burgdorferi, Ehrlichia* spp., *Anaplasma* spp., and *Dirofilaria immitis*. Abdominal ultrasound identified moderate mesenteric lymphadenopathy and regionally hyperechoic mesentery. Joint fluid cytology raised concern for large cell lymphoma. Flow cytometry identified 37% aberrant large CD4+ T cells, suggesting lymphoma. PCR for antigen receptor rearrangement (PARR) testing on the joint fluid confirmed clonal expansion of T cells, further supporting T‐cell lymphoma. Peripheral blood PCR was positive for *Anaplasma phagocytophilum* . The dog's clinical signs and laboratory abnormalities resolved after a 30‐day treatment with doxycycline. After treatment, joint fluid analysis, including cytology and flow cytometry, was normal. This case highlights the importance of considering *A. phagocytophilum* as a differential diagnosis in dogs with a clonal expansion of CD4+ T cells, emphasizing the comprehensive diagnostic approach.

**Abbreviations**
GA: granulocytic anaplasmosis
FC: flow cytometry
PARR: PCR for antigen receptor rearrangement

## 1 Introduction

*Anaplasma phagocytophilum* is the causative agent of granulocytic anaplasmosis (GA), a common bacterial vector‐borne infection diagnosed in dogs across the United States. Endemic regions within the United States mirror the distribution of *Ixodes* spp. of ticks, encompassing the upper Midwest, Northeast, and to a lesser extent, the West Coast. *A. phagocytophilum* is an obligate intracellular bacterium that infects granulocytes, primarily neutrophils [1]. Numerous genetic variants of *A. phagocytophilum* are identified globally, though the clinical presentation remains consistent [2, 3, 4]. The most common signs include fever, lethargy, and inappetence but can also include vomiting, diarrhea, and inflammatory polyarthropathy [5]. Peripheral blood cell abnormalities most commonly include thrombocytopenia, anemia, and neutropenia [1, 4].

ELISA point‐of‐care tests, peripheral blood smear cytology, immunofluorescent antibody (IFA), and PCR are commonly used to diagnose *A. phagocytophilum* . ELISA testing has a high sensitivity, but low specificity [6]. It relies on antibody detection, which can lead to false negatives early in the course of infection. Additionally, antibodies can circulate for many months after infection; therefore, a positive ELISA does not confirm an active infection. Cytology is specific when morulae are identified, but is an insensitive method of diagnosis [1, 4]. PCR testing is highly sensitive and specific in dogs, but false negative PCR testing can occur in late‐stage infections or in dogs on antibiotic therapy at the time of sample collection [6, 7].

Some vector‐borne diseases rarely induce a monoclonal gammopathy or clonal expansion of T lymphocytes in dogs [8, 9, 10]. Monoclonal gammopathies with naturally occurring *Ehrlichia canis*, Leishmaniasis, and *Bartonella* infections have been reported that are indistinguishable from dogs with multiple myeloma [8, 10, 11]. *Ehrlichia canis* infection can also be associated with a clonally rearranged T‐cell receptor gene using PCR [12]. In these cases, dogs can be mistakenly diagnosed with round cell neoplasia, which carries a much more guarded prognosis and different treatment plan. Flow cytometry (FC) and PCR for antigen receptor rearrangement (PARR) are tests used to differentiate between benign infectious/inflammatory diseases and round cell malignancies. Flow cytometry uses fluorochrome‐labeled antigen–antibody complexes bound to cells, enabling the identification of specific cell types and markers. For diagnosis of lymphoma, the sensitivity and specificity of FC have been reported to range from 67.6% to 100% and 92.6% to 100%, respectively [13, 14]. In contrast, PARR utilizes PCR of B or T cell receptor genes to determine clonality by using genomic DNA and PCR primers that are specific to the canine V(D)J splice junctions of B and T cell receptor gene segments in lymphocytes [12, 15]. The primary application of PARR in veterinary medicine is the confirmation or differentiation of malignant neoplasia by detecting genetically identical populations of lymphocytes when cytology or histopathology yields inconclusive results. The sensitivity and specificity of PARR in veterinary medicine range from 67% to 100% and 94% to 100%, respectively [13, 15, 16, 17]. This case report describes a dog with PCR confirmed *A. phagocytophilum* that simultaneously had both FC and PARR reported T cell lymphoma on joint fluid analysis.

## 2 Case Description

A 4‐year‐old spayed female Golden Retriever in Minnesota was examined for evaluation of a two‐day history of lethargy, anorexia, and hindlimb lameness. Approximately 2 weeks before presentation, numerous ticks had been removed from the dog after it had traveled to a cabin in Northern Minnesota. No other travel history outside of Minnesota was reported. Physical examination identified a fever of 103.2°F (39.6°C) and stiff hind limb gait. The complete blood count (CBC) showed thrombocytopenia (99 K/μL, reference interval [RI] 143–448 K/μL) and lymphopenia (893/μL, RI 1060–4950/μL). Serum biochemistry revealed hypoalbuminemia (2.4 g/dL, RI 2.7–3.9 g/dL) but was otherwise normal. Three‐view abdominal radiographs identified possible decreased retroperitoneal detail. A SNAP 4Dx test (IDEXX Laboratories, Westbrook, ME) was negative for *Borrelia burgdorferi, Ehrlichia* spp., *Anaplasma* spp., and *Dirofilaria immitis*. The dog was prescribed oclacitinib (0.56 mg/kg PO Q12h) and amoxicillin‐clavulanic acid (13.1 mg/kg PO Q12h) for pruritus and suspected bacterial pyoderma, and carprofen (1.75 mg/kg PO Q12h) for joint pain and fever. Due to continued lameness and lethargy, the dog was referred to a local emergency and specialty hospital the next day for further evaluation, and these medications were discontinued after a single dose.

Physical examination at presentation to the referral hospital identified a persistent fever of 104.8°F (40.4°C), pain on tarsal extension bilaterally, and difficulty rising in all four limbs. A SNAP 4Dx was repeated with no positive results. CBC confirmed thrombocytopenia (76 K/μL, RI 143–448 K/μL), lymphopenia (880/μL, RI 1050–5100/μL), and eosinopenia (10/μL, RI 60–1230/μL). A blood smear reviewed by a board‐eligible pathologist confirmed a moderately decreased platelet count with mostly large to occasionally giant platelets. The manual platelet count was estimated to be 75–125 K/μL. No hemoparasites were identified on the blood smear evaluation. A urinalysis showed concentrated urine (specific gravity > 1.050) and a quiet sediment with suspected presence of cocci (Sedivue DX, IDEXX Laboratories). The dog was admitted for stabilization with intravenous administration of isotonic fluids (80 mL/kg/day), doxycycline (6.7 mg/kg PO Q12h) and maropitant (1 mg/kg IV Q24h).

The next day an abdominal ultrasonographic examination was performed by a board‐certified veterinary radiologist. Mildly to moderately enlarged, hypoechoic mesenteric lymph nodes (9 mm thick) and mildly hyperechoic mesentery were reported. A canine vector‐borne disease PCR panel testing for *Anaplasma phagocytophilum, Anaplasma platys, Babesia canis, Babesia* sp. *(non‐canis), Bartonella henselae, Bartonella vinsonii, Ehrlichia canis, Ehrlichia* spp. *(non‐canis), Mycoplasma haemocanis/haematoparvum, Neorickettsia risticii, and Rickettsia rickettsii* (Antech Diagnostics, Fountain Valley, CA) was submitted after a single dose of doxycycline administered orally 12 h earlier. Joint fluid was collected from the right carpus, left carpus, and left tarsal joints. The joint fluid was mildly turbid and had subjectively reduced viscosity. Cytological analysis identified moderate to marked numbers of pleomorphic mononuclear cells, which were consistent across all joint samples. A leukocyte differential on the right carpal fluid showed 25% neutrophils, 4% small mononuclear cells, and 71% large mononuclear cells raising concern for large cell lymphoma. Total nucleated cell counts on the joint fluid samples were not performed. For additional clarification, the joint effusion was submitted for FC. The dog was discharged home roughly 24 h after admission with improving clinical signs after beginning oral doxycycline (6.82 mg/kg Q12h) therapy alone. Additional medications to address the dog's anorexia and arthralgia were not needed at the time of discharge as those signs were resolving.

Several days later, FC results from the joint fluid identified an aberrant population of 37% large CD4+ T lymphocytes, 0% CD21+ B lymphocytes, and 57% neutrophils (CD4+/CD5‐). The remainder of the cells (6%) were dead and unable to be classified. Interpretation of the FC results by the clinical pathologist was consistent with T cell lymphoma. For additional confirmation, the joint fluid was submitted for PARR. TCRγ PARR results confirmed a clonal expansion of T cells, supporting a diagnosis of T cell lymphoma. While PARR results were pending, the vector‐borne PCR panel returned positive for *Anaplasma phagocytophilum* . All other PCR results were negative. The dog's owner reported continued clinical improvement with doxycycline alone. Given the history, positive clinical response, and confirmed *A. phagocytophilum* PCR result, doxycycline was extended to a 30‐day course, and lymphoma treatment was deferred.

**FIGURE 1:**
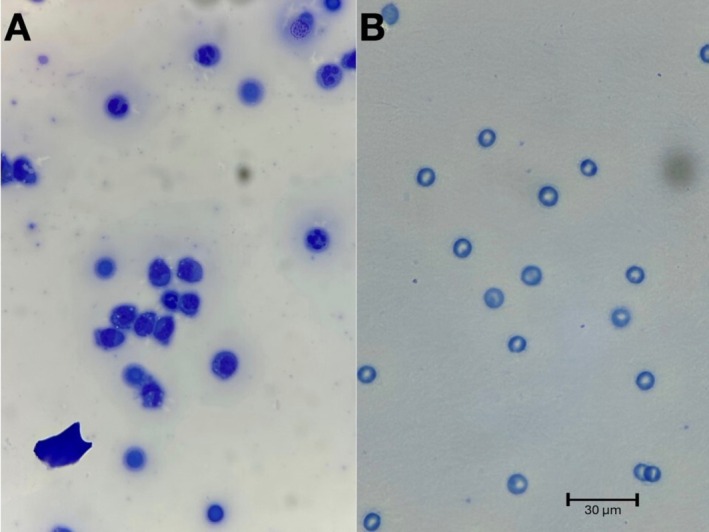
(A) Joint fluid cytology before treatment with doxycycline; (B) joint fluid cytology after 4‐week treatment with doxycycline.

Twenty‐eight days after hospitalization, the dog was examined and was clinically normal. The dog's fever, thrombocytopenia, and clinical signs associated with polyarthropathy had resolved. Abdominal ultrasonography identified improving mesenteric lymphadenopathy and normal mesentery. Synovial fluid was collected from the right carpus, left carpus, and left tarsal joints and submitted for cytology and FC. Both results showed resolution of the previously identified aberrant T cells (Figure 1). Doxycycline therapy was continued for a total of 30 days. The dog was reported to be clinically normal 2 years after completion of doxycycline treatment, when the last follow‐up was performed.

## 3 Discussion

Granulocytic anaplasmosis is a frequent consideration in dogs exhibiting lethargy, fever, and clinical signs of polyarthropathy in endemic areas. The diagnosis, however, is often complicated by the shared clinical manifestations with many other infectious, inflammatory, and neoplastic conditions and the variable sensitivity and specificity of available tests. While point‐of‐care ELISA tests are readily available and cost‐effective, they cannot confirm active infection and exhibit reduced sensitivity within the first four days of clinical *A. phagocytophilum* infection [7]. Clinical signs of *A. phagocytophilum* occur 1–2 weeks after infection. Antibodies can appear as early as 8 days after infection, but titers are often low during this acute phase [1]. In this dog, the time from tick exposure to clinical signs was approximately 2 weeks. The SNAP 4Dx was performed at about 2.5 weeks after exposure and was negative. While often the ELISA is positive as early as 8 days after infection, antibody concentrations can be low in this stage of the disease resulting in a false negative test. This was the case in our dog. Consequently, additional diagnostics, such as blood smear cytology, acute and convalescent antibody titers, or PCR, are typically required for confirmation [1, 4].

Lameness and polyarthritis are common clinical signs with GA; yet, few studies document the results of joint fluid evaluation in these dogs. Joint fluid was evaluated in 5 of 63 *A. phagocytophilum* positive dogs and found abnormal joint fluid color and viscosity, increased protein concentration, and elevated cell counts consisting predominantly of neutrophils [5]. In 3 of 5 dogs, PCR for *A. phagocytophilum* was performed on the joint fluid, and there were positive results in 2 of the 3 dogs. In another study of 18 dogs with GA, joint fluid evaluation was reported in one out of the 18 dogs with signs of polyarthritis [18]. The joint fluid contained > 90% neutrophils, and an aerobic culture of the joint fluid was negative. With limited reports available detailing the composition of joint fluid in dogs with active GA, it remains uncertain whether a predominantly neutrophilic joint fluid cytology is typical or whether a mononuclear population, like the one reported in this case, could be more common than anticipated and simply not yet described.

In this case, joint cytology results raised concern for large cell lymphoma despite the remainder of the clinical picture strongly suggesting GA. This prompted submission of FC and PARR. Flow cytometry and PARR are commonly used for the diagnosis and staging of lymphoma and other lymphoproliferative disorders in dogs. Both tests screen for clonal populations of leukocytes. When clonality is identified, this is typically unquestionably diagnostic of malignancy. There are reported instances of monoclonal gammopathies associated with Ehrlichiosis, Leishmaniasis, and Bartonellosis in dogs and clonal T cell expansion detected by PARR with *Leishmania infantum, Ehrlichia canis*, and in a single dog with Panola Mountain *Ehrlichia* spp. [8, 9, 10, 19, 20, 21] Our dog had a clonal population of aberrant CD4+ T cells in joint fluid with both FC and PARR with PCR confirmed GA. A similar case is reported describing a clonal T cell expansion in a man with Anaplasmosis mimicking lymphoma [22]. While our dog did not have testing performed to screen for *Leishmania infantum*, this disease is not found in Minnesota and the dog had no history of travel outside of this state, making a co‐infection with this pathogen unlikely to be the cause of the T cell expansion. *Ehrlichia* co‐infection was excluded in this dog based on a negative blood smear evaluation, negative titer, and negative PCR result. Bartonellosis was considered unlikely due to the negative PCR result, the dog's geographic location, and vector exposure [23]. The T cell clonality was then confirmed to have resolved with repeat FC analysis on the joint fluid after a 30‐day course of doxycycline. The expansion of T lymphocytes in these particular vector‐borne diseases is not surprising given the intracellular location of these pathogens, which will induce a cell‐mediated immune response. The pathogenesis of the clonal expansion of lymphocytes in these select infections is uncertain.

In conclusion, this case report highlights the importance of performing a thorough history and clinical evaluation in all dogs with non‐specific signs attributable to a diverse group of diseases. In dogs with suspected infectious diseases, recognition of the limitations of in‐house antibody‐based testing should prompt clinicians to pursue confirmatory testing if initial screening testing is negative.

## Disclosure

Authors declare no off‐label use of antimicrobials.

## Ethics Statement

Authors declare no institutional animal care and use committee or other approval was needed. Authors declare human ethics approval was not.

## Conflicts of Interest

The authors declare no conflicts of interest.
